# Amlodipine increases risk of primary open-angle glaucoma

**DOI:** 10.1186/s40885-024-00290-9

**Published:** 2024-11-01

**Authors:** Steven Lehrer, Peter H. Rheinstein

**Affiliations:** 1 Department of Radiation Oncology, Icahn School of Medicine at Mount Sinai, New York, NY USA; 2 Severn Health Solutions, Severna Park, MD USA

**Keywords:** Epidemiology, Glaucoma, Intraocular pressure, Systemic medication

## Abstract

**Background:**

The use of calcium channel blockers is associated with primary open-angle glaucoma (POAG) in a statistically meaningful but minor way. In general, those who had received calcium channel blocker medication were at a 23% increased risk of developing glaucoma in comparison to those who had never taken the antihypertensive drugs. We wished to confirm this association and examine POAG genes that might be involved, since the genetics has not yet been analyzed.

**Methods:**

We used MedWatch and UK Biobank data to evaluate the effects of amlodipine on POAG and intraocular pressure (IOP). We analyzed three POAG-associated single-nucleotide polymorphisms: rs9913911, an intron variant in growth arrest-specific 7 (GAS7), one of the genes that influences IOP; rs944801, an intron variant within CDKN2B-AS1, and rs2093210, an intron variant within SIX6, known to be associated with vertical cup-disc ratio, an important optic nerve head parameter that is often used to define or diagnose glaucoma.

**Results:**

Amlodipine use in MedWatch doubled the prevalence of POAG, from 0.0805 to 0.177%, a small but significant increase. Multivariate analysis by logistic regression of UK Biobank data revealed that POAG risk was significantly increased with age, male sex, major alleles of rs9913911 (GAS7) and rs944801 (CDKN2B-AS1), and minor allele of rs2093210 (SIX6). Amlodipine increased POAG risk by 16.1% (*P* = 0.032). Amlodipine has not been associated with increased IOP. We confirmed this lack of association and in addition found that GAS7, associated with IOP, was not associated with POAG risk and amlodipine. But CDKN2B-AS1 and SIX6, POAG genes not associated with IOP, were associated with POAG and amlodipine.

**Conclusions:**

Amlodipine, a frequently prescribed drug and first line treatment for hypertension, has a potentially hazardous relationship with POAG. Knowledge of this link can guide the prescribing of alternate drugs for hypertensive individuals who have glaucoma or are at risk for it. Diuretics and β-blockers are not associated with POAG or increased IOP and could be substituted for amlodipine in hypertensive patients at risk POAG.

**Trial registration:**

None.

**Graphical Abstract:**

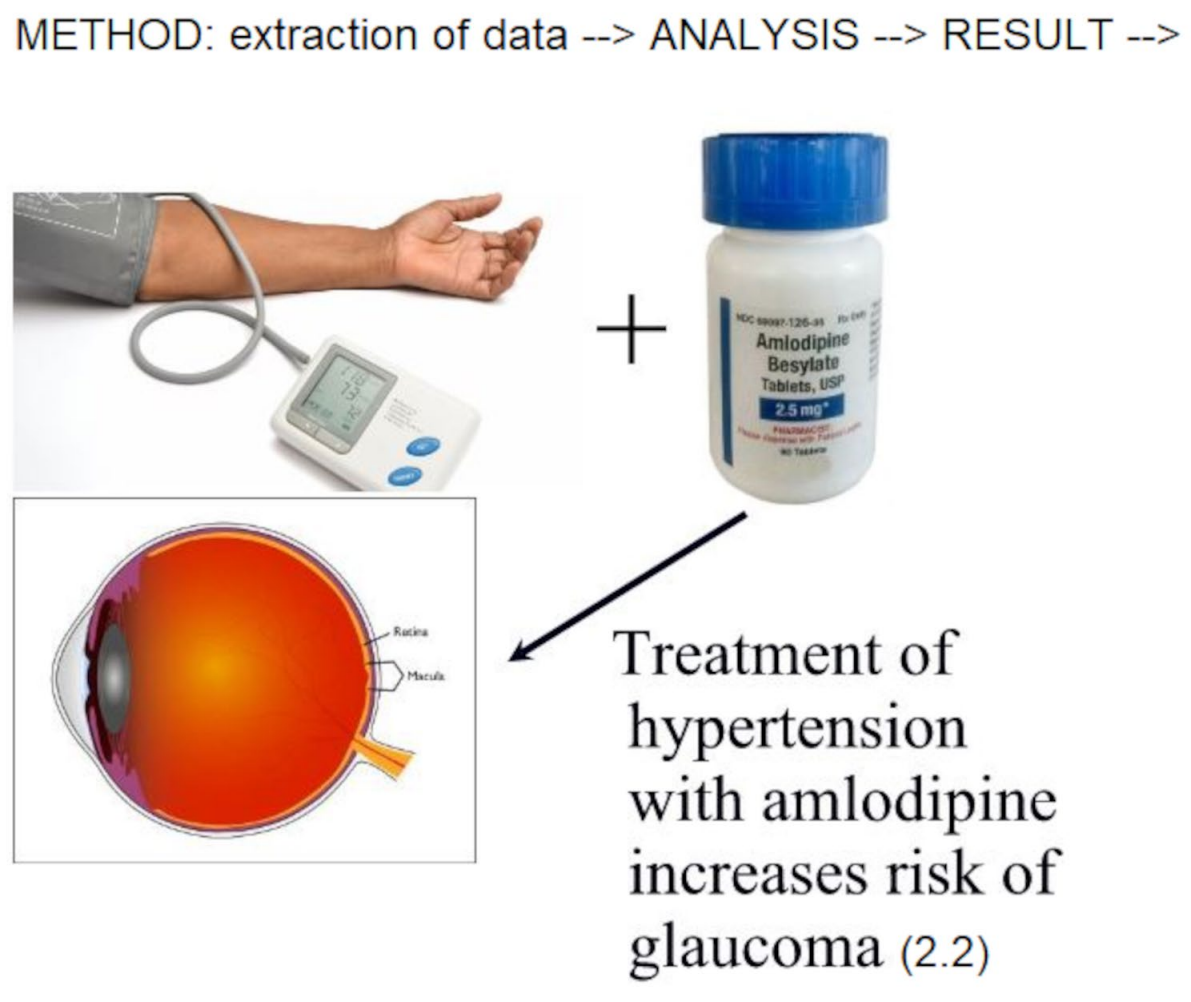

## Background

According to a large meta-analysis [1], the use of calcium channel blockers (CCBs) was associated with glaucoma in a statistically meaningful but minor way. In general, those who had received CCB medication were at a 23% increased risk of developing glaucoma in comparison to those who had never taken the antihypertensive drugs. A previous study of US insurance billing data [2] found that CCBs were associated with a 26% glaucoma increase.

Amlodipine is the most prescribed CCB and a first line agent for hypertension. Amlodipine, a dihydropyridine which is vascular selective, inhibits calcium influx by blocking voltage-dependent L-type calcium channels. Amlodipine has the longest half-life (30–50 h) when compared to nifedipine and other CCBs. The advantage of having a half-life this long is that dosage (5–10 mg) can be once a day. The US Food and Drug Administration (FDA) first approved amlodipine besylate in 1987 [3, 4]. New research [5] indicates that amlodipine is unlikely to contribute to significant heart failure risk.

In the current study, we used MedWatch and UK Biobank data to analyze the effects of amlodipine on primary open-angle glaucoma (POAG) and intraocular pressure (IOP).

## Methods

We made use of information from the FDA’s Adverse Event Reporting Program and Safety Information, MedWatch [6, 7]. In 1993, MedWatch was established to gather information about unfavorable incidents in the medical field. Any unpleasant experience connected to the use of a medicinal substance is called an adverse event. Reports of adverse reactions and quality issues are gathered by the MedWatch system, mostly for medications and medical equipment but also for other FDA-regulated items (e.g., nutritional supplements, cosmetics, medical foods, and infant formulae).

A public database contains machine-readable MedWatch data, including manufacturer reports of adverse medication reactions. To query the database, we utilized the web application OpenVigil 2.1 [8, 9]. Data for OpenVigil comes only from MedWatch and the FDA; it does not come from social media [10]. Using the criteria of Evans et al. [11], OpenVigil computes proportional reporting ratios (PRRs) from adverse drug reaction data to ascertain whether the drug and adverse event together are connected. “PRR = 2” means that compared to the general population, drug users experience adverse reactions twice as frequently. In accordance with the criterion of Evans et al. [11], the adverse reaction and the medication are linked if there are more than three adverse events, a chi-squared > 4 (*P* = 0.05), and a PRR > 2.

The UK Biobank is a large prospective observational study of men and women with no link to MedWatch. Participants were recruited from across 22 centers located throughout England, Wales, and Scotland between 2006 and 2010 and continue to be longitudinally followed for capture of subsequent health events [12]. This methodology is like that of the ongoing Framingham Heart Study [13], with the exception that the UK Biobank program collects postmortem samples, which Framingham did not.

Our UK Biobank application was approved as UK Biobank project 57,245 (SL, PHR). Our analysis included all subjects with POAG. POAG diagnosis was ascertained using the International Classification of Diseases, 10th Revision (ICD-10), H40.1. Age at glaucoma diagnosis was from data field 4689.

The IOP was measured once for each eye (right eye first) using the ORA (Reichert Corp), and only one measurement per eye was taken. Participants who had eye surgery within the previous 4 weeks or those with possible eye infections were precluded from having IOP measured. The ORA flattens the cornea with a jet of air and uses an electro-optical system to measure the air pressures at which the cornea flattens both inward and outward. The average of the two ORA pressure values was calibrated against Goldmann applanation tonometer measures to derive IOP. The IOP corneal corrected was derived using proprietary formulae to correct for corneal biomechanical properties [14]. Corneal hysteresis was not measured. We analyzed three POAG-associated single-nucleotide polymorphisms (SNPs) (Table 1). The three genes and three SNPs were chosen for analysis because the three SNPs, rs9913911, rs944801, rs2093210, are well characterized, associated with POAG, and were available in the UK Biobank data.

**Table 1:** SNPs analyzed in this study

Gene	Chromosome	SNP	MajA	MA	MAF	MSC
GAS7	17p13.1	rs9913911	A	G	0.44	Intron variant
CDKN2B-AS1	9p21.3	rs944801	C	G	0.41	Intron variant
SIX6	14q23.1	rs2093210	T	C	0.34	Intron variant

SNP, single-nucleotide polymorphism; MajA, major allele; MA, minor allele; MAF, minor allele frequency; MSC, most severe consequence; GAS7, growth arrest-specific 7

Data processing was performed on Minerva, a Linux mainframe with Centos 7.6, at the Icahn School of Medicine at Mount Sinai (New York, NY, USA). We used PLINK, a whole-genome association analysis toolset, to analyze the UK Biobank chromosome files [15] and the UK Biobank Data Parser (ukbb_parser), a python-based package that allows easy interfacing with the large UK Biobank dataset [16]. Statistical analysis was done with IBM SPSS ver. 26.0 (IBM Corp).

## Results

The mean age of 502,494 UK Biobank subjects was 56 ± 8 years; 54% were female, 46% were male, and 95% were white British. A total of 633 subjects had POAG.

MedWatch data of 11,439,756 subjects to evaluate the criteria of Evans et al. [11] for POAG and amlodipine are in Table 2 (chi-squared, 213; PRR, 2.2; 95% confidence interval [CI], 2–2.5). According to the criteria (*n* > 3 adverse events, chi-squared > 4, PRR > 2) [11], amlodipine use is related to POAG. Amlodipine use doubled the prevalence of POAG, from 0.0805 to 0.177%, a small but significant increase.

**Table 2:** MedWatch data to evaluate the criteria of Evans et al. [11] for POAG

Variable	Amlodipine	All other drugs	Total
POAG	340	9,051	9,391
All other adverse event	191,720	11,238,645	11,430,365
Total	192,060	11,247,696	11,439,756
POAG (%)	0.1770	0.0805	-

POAG, primary open-angle glaucoma

Chi-squared, 213; proportional reporting ratio, 2.2 (95% confidence interval, 2–2.5). According to the criteria of Evans et al. [11] (*n* > 3 adverse events, chi-squared > 4, proportional reporting ratio > 2), amlodipine use is related to POAG

IOP corneal corrected right eye and left eye is in Table 3. There was no significant effect of amlodipine on IOP right eye (*P* = 0.740) or left eye (*P* = 0.798).

**Table 3:** Intraocular pressure corneal compensated left eye and right eye

Amlodipine	No. of subjects	Mean	Standard deviation
Left eye			
No	611	18.98	5.96
Yes	58	18.94	7.66
Right eye			
No	610	18.95	5.31
Yes	59	18.86	5.28

There was no significant effect of amlodipine on intraocular pressure right eye (*P* = 0.740) or left eye (*P* = 0.798)

Alleles of rs9913199 (growth arrest-specific 7, GAS7) in subjects without/with POAG are in Table 4. The major allele A was associated with increased POAG risk with amlodipine (51.7% vs. 46.2%) but the association was not significant (two-tailed Fisher exact test, *P* = 0.222). The rs944801 alleles (CDKN2B-AS1) in subjects without/with POAG are in Table 5. The major allele C was significantly associated with amlodipine in POAG (54.2% vs. 42.8%, *P* = 0.004). The rs2093210 alleles (SIX6) in subjects without/with POAG are in Table 6. The minor allele C was significantly associated with amlodipine in POAG (32.5% vs. 22.3, *P* = 0.001).

**Table 4:** The rs9913199 (GAS7) in subjects without/with POAG

rs9913911 (GAS7)	Amlodipine			*P*-value
	No	Yes	Total	
Without POAG				< 0.001
AA				
Count	181,447	8,358	189,805	
Within amlodipine (%)	39.6	41.2	39.7	
AG				
Count	213,180	9,138	222,318	
Within amlodipine (%)	46.6	45.0	46.5	
GG				
Count	63,251	2,808	66,059	
Within amlodipine (%)	13.8	13.8	13.8	
Total				
Count	457,878	20,304	478,182	
Within amlodipine (%)	100	100	100	
With POAG				0.222
AA				
Count	1,357	120	1,477	
Within amlodipine (%)	46.2	51.7	46.6	
AG				
Count	1,277	88	1,365	
Within amlodipine (%)	43.5	37.9	43.1	
GG				
Count	303	24	327	
Within amlodipine (%)	10.3	10.3	10.3	
Total				
Count	2,937	232	3,169	
Within amlodipine (%)	100	100	100	

The major allele A was associated with increased POAG risk with amlodipine (51.7% vs. 46.2%) but the association was not significant (two-tailed Fisher exact test, *P* = 0.222)

GAS7, growth arrest-specific 7; POAG, primary open-angle glaucoma

**Table 5:** The rs944801 (CDKN2B-AS1) in subjects without/with POAG

rs944801 (CDKN2B-AS1)	Amlodipine			*P*-value
	No	Yes	Total	
Without POAG				< 0.001
CC				
Count	161,063	7,683	168,746	
Within amlodipine (%)	34.8	37.5	35.0	
CG				
Count	220,334	9,460	229,794	
Within amlodipine (%)	47.7	46.2	47.6	
GG				
Count	80,855	3,349	84,204	
Within amlodipine (%)	17.5	16.3	17.4	
Total				
Count	462,252	20,492	482,744	
Within amlodipine (%)	100	100	100	
With POAG				0.004
CC				
Count	1,269	130	1,399	
Within amlodipine (%)	42.8	54.2	43.6	
CG				
Count	1,344	86	1,430	
Within amlodipine (%)	45.3	35.8	44.6	
GG				
Count	355	24	379	
Within amlodipine (%)	12.0	10.0	11.8	
Total				
Count	2,968	240	3,208	
Within amlodipine (%)	100	100	100	

The major allele C was associated with amlodipine in POAG (54.2% vs. 42.8%, *P* = 0.004)

POAG, primary open-angle glaucoma

**Table 6:** rs2093210 (SIX6) in subjects without/with POAG

rs2093210 (SIX6)	Amlodipine			*P*-value
	No	Yes	Total	
Without POAG				< 0.001
TT				
Count	165,226	7,080	172,306	
Within amlodipine (%)	35.7	34.6	35.7	
TC				
Count	217,522	9,195	226,717	
Within amlodipine (%)	47.0	44.9	46.9	
CC				
Count	79,706	4,202	83,908	
Within amlodipine (%)	17.2	20.5	17.4	
Total				
Count	462,454	20,477	482,931	
Within amlodipine (%)	100	100	100	
With POAG				0.001
TT				
Count	885	72	957	
Within amlodipine (%)	29.8	30.0	29.8	
TC				
Count	1,423	90	1,513	
Within amlodipine (%)	47.9	37.5	47.1	
CC				
Count	663	78	741	
Within amlodipine (%)	22.3	32.5	23.1	
Total				
Count	2,971	240	3,211	
Within amlodipine (%)	100	100	100	

The minor allele C was associated with amlodipine in POAG (32.5% vs. 22.3, *P* = 0.001)

POAG, primary open-angle glaucoma

Table 7 contains results of multivariate analysis (logistic regression). POAG risk was significantly increased with age, male sex, major alleles of rs9913911 (GAS7) and rs944801 (CDKN2B-AS1), and minor allele of rs2093210 (SIX6). Amlodipine increased POAG risk by 16.1% (odds ratio [OR], 1.161; *P* = 0.032).

**Table 7:** Multivariate analysis (logistic regression)

POAG-dependent variable	Odds ratio	95% Confidence interval	*P*-value
Male sex	1.234	1.150–1.324	< 0.001
Age (yr)	1.122	1.115–1.128	< 0.001
rs9913911 (GAS7)	0.791	0.751–0.834	< 0.001
rs944801 (CDKN2B-AS1)	0.727	0.689–0.766	< 0.001
rs2093210 (SIX6)	1.273	1.212–1.336	< 0.001
Amlodipine	1.161	1.014–1.329	0.032

POAG risk was significantly increased with age, male sex, major alleles of rs9913911 (GAS7) and rs944801 (CDKN2B-AS1), and minor allele of rs2093210 (SIX6). Amlodipine increased POAG risk by 16.1% (odds ratio, 1.161)

POAG, primary open-angle glaucoma; GAS7, growth arrest-specific 7

## Discussion

We found a small but statistically significant correlation between amlodipine use and glaucoma. People who had taken amlodipine were at a 16.1% increased risk of POAG in comparison to those who had never taken amlodipine.

Of the three SNPs we analyzed, rs9913911 is an intron variant in GAS7, one of the genes that influences IOP, found in a chromosomal area previously identified by a glaucoma linkage study and subsequently by a genome-wide association study [17]. GAS7, at chromosome 17p31.1, is quite close to a cannabis receptor associated with IOP at chromosome 17p31.3 [18, 19], and has been linked with schizophrenia in a Chinese population [20]. The rs944801, an intron variant within CDKN2B-AS1, and rs2093210, an intron variant within SIX6, are known to be associated with vertical cup-disc ratio, an important optic nerve head parameter that is often used to define or diagnose glaucoma [21].

Amlodipine has not been associated with increased IOP [22]. We confirmed this lack of association and in addition found that GAS7, associated with IOP, was not associated with POAG risk and amlodipine. But CDKN2B-AS1 and SIX6, POAG genes not associated with IOP, were associated with POAG and amlodipine.

Amlodipine and other CCBs lower systemic blood pressure by blocking the function of calcium channels on the myocardium, adrenal cortical cells, and vascular smooth muscle. There have been conflicting reports regarding the use of CCBs, IOP, and POAG risk [23]. According to certain research, CCBs may enhance choroidal and optic nerve head blood flow and slow the degradation of the visual field in open-angle glaucoma patients. According to in vitro research, calcium channel blocking lessens the extracellular matrix gene response that mechanical strain induces in lamina cribrosa cells. According to some, CCBs may cause venous dilatation, which would improve perfusion to the optic nerve head. But according to another research, using CCBs is linked to a higher risk of POAG.

Zheng et al. [24] analyzed US health insurance data in a case-control design and found a similar correlation between POAG and the use of CCBs (OR, 1.26; 95% CI, 1.18–1.35). Following the correction for other drugs, such as systemic β-blockers (OR, 1.23; 95% CI, 1.14–1.33), the association remained statistically significant. Kastner et al. [22] reported CCB use was associated with glaucoma and related traits among UK Biobank participants. The usage of CCBs and the prevalence of glaucoma were found to be correlated by Asefa et al. [25] and Langman et al. [26]. The retina may be directly affected by CCBs; in the past, CCB use was linked to a thinner layer of macular retinal nerve fiber and a thinner layer of ganglion cell–inner plexiform layer.

### Limitations

First, there are errors in the MedWatch data, including underreporting and overreporting, missing denominators (doses for a drug), and incorrect, duplicate, or missing data in the database [8]. Because of this, the overall number of OpenVigil reports of adverse events for all drugs and/or the drug in question may differ slightly for various drugs and for adverse events connected to the same drug. The problematic MedWatch data have caused all analytical software applications, including OpenVigil, to deal with this challenge [27]. FDA has released warnings on MedWatch: reports are not useful for establishing rates of occurrence. It is not appropriate to gauge the probability of a side effect happening based on the quantity of suspected reactions recorded in the FDA Adverse Event Reporting System. Not every adverse event or medication error involving a product is reported to the FDA. Numerous factors, like the length of time a product has been promoted and publicity surrounding an event might affect whether it is reported. As a result, it is not possible to calculate the incidence (occurrence rates) of the reactions mentioned using the data in these reports. It is important to note that the FDA Adverse Event Reporting System data does not, by itself, represent the drug’s safety profile (https://www.fda.gov/drugs/surveillance/questions-and-answers-fdas-adverse-event-reporting-system-faers).

Another limitation of our investigation stemmed from the way in which the UK Biobank determines cases of glaucoma through a combination of linked ICD codes and self-report. Our basic case definition (based solely on ICD codes) may be specific, but it might miss a significant percentage of actual patients with glaucoma who might not be included in a database kept at a hospital. While self-report may identify a greater number of patients, it carries the potential of recollection bias and/or misclassification.

Third, we were unable to examine the length of time amlodipine was taken or amlodipine dosage, which could be crucial in understanding its connection to glaucoma.

Fourth, CCB is a heterogeneous class which includes dihydropyridines and nondihydropyridines. We analyzed only one drug of the dihydropyridine group, amlodipine.

Lastly, it would be worthwhile to compare amlodipine to other CCBs and other antihypertensive agents.

## Conclusions

Amlodipine, an often-prescribed drug and first line treatment for hypertension, has a potentially hazardous relationship with POAG. Knowledge of this link can guide the prescribing of alternate drugs for hypertensive individuals who have glaucoma or are at risk for it. Diuretics and β-blockers are not associated with POAG or increased IOP [1] and could be substituted for amlodipine in hypertensive patients with POAG. β-Blockers are known to decrease IOP. Eyedrops containing the β-blocker timolol (Timoptic, Bausch + Lomb) and combinations of timolol with dorzolamide (Cosopt, Théa) as well as timolol with brimonidine (Combigan, Allergan) are FDA approved for treatment of glaucoma. Other medications also reduce risk of glaucoma [23].

The risk of POAG associated with amlodipine is probably too low to recommend regular follow up eye examinations with amlodipine usage in most cases. The risk appears to be limited to a small population with specific genes. Therefore, an ophthalmological examination before amlodipine administration to all patients is probably not necessary. But hypertensive patients with a family history of glaucoma or other risk factors should have at least an initial eye examination before amlodipine is begun.

## Data Availability

Data sources described in this article are publicly available at MedWatch (http://h2876314.stratoserver.net:8080/OV2/search/) or after an application approval to UK Biobank (https://www.ukbiobank.ac.uk/).

## Abbreviations

CCB: Calcium channel blocker
CI: Confidence interval
FDA: US Food and Drug Administration
GAS7: Growth arrest-specific 7
ICD-10: International Classification of Diseases, 10th Revision
IOP: Intraocular pressure
OR: Odds ratio
POAG: Primary open-angle glaucoma
PRR: Proportional reporting ratio
SNP: Single-nucleotide polymorphism
